# Biofilms in Diabetic Foot Ulcers: Significance and Clinical Relevance

**DOI:** 10.3390/microorganisms8101580

**Published:** 2020-10-14

**Authors:** Cassandra Pouget, Catherine Dunyach-Remy, Alix Pantel, Sophie Schuldiner, Albert Sotto, Jean-Philippe Lavigne

**Affiliations:** 1 Virulence Bactérienne et Maladies Infectieuses, INSERM U1047, Université de Montpellier, 30908 Nîmes, France; cassandra.pouget@gmail.com; 2 Virulence Bactérienne et Maladies Infectieuses, INSERM U1047, Université de Montpellier, Service de Microbiologie et Hygiène Hospitalière, Clinique du Pied Gard Occitanie, CHU Nîmes, 30029 Nîmes, France; catherine.remy@chu-nimes.fr (C.D.-R.); alix.pantel@chu-nimes.fr (A.P.); 3 Virulence Bactérienne et Maladies Infectieuses, INSERM U1047, Université de Montpellier, Service des Maladies Métaboliques et Endocriniennes, Clinique du Pied Gard Occitanie, CHU Nîmes, 30240 Le Grau du Roi, France; sophie.schuldiner@chu-nimes.fr; 4 Virulence Bactérienne et Maladies Infectieuses, INSERM, Université de Montpellier, Service des Maladies Infectieuses et Tropicales, Clinique du Pied Gard Occitanie, CHU Nîmes, 30029 Nîmes, France; albert.sotto@chu-nimes.fr

**Keywords:** biofilm, commensal bacteria, diabetic foot infection, diabetic foot ulcer, pathogenic bacteria, pathogroups

## Abstract

Foot infections are the main disabling complication in patients with diabetes *mellitus*. These infections can lead to lower-limb amputation, increasing mortality and decreasing the quality of life. Biofilm formation is an important pathophysiology step in diabetic foot ulcers (DFU)—it plays a main role in the disease progression and chronicity of the lesion, the development of antibiotic resistance, and makes wound healing difficult to treat. The main problem is the difficulty in distinguishing between infection and colonization in DFU. The bacteria present in DFU are organized into functionally equivalent pathogroups that allow for close interactions between the bacteria within the biofilm. Consequently, some bacterial species that alone would be considered non-pathogenic, or incapable of maintaining a chronic infection, could co-aggregate symbiotically in a pathogenic biofilm and act synergistically to cause a chronic infection. In this review, we discuss current knowledge on biofilm formation, its presence in DFU, how the diabetic environment affects biofilm formation and its regulation, and the clinical implications.

## 1. Introduction

People suffering from diabetes *mellitus* have a 15–25% lifetime incidence of developing a diabetic foot ulcer (DFU) [1]. Infection is the most common, severe, and costly complication of diabetes *mellitus* [2], with high risk of mortality and morbidity due to lower limb amputation [3]. Wound infection, faulty wound healing, and ischemia are the most common precursors to diabetes-related amputations. Indeed, 80% of lower-limb amputations in diabetic patients are preceded by biofilm infected foot ulceration [4,5]. Infected wounds result in an increased risk of death within 18 months [6]. The host–microorganism interface plays a major role in DFU development. In DFU, bacteria are classically organized into functionally equivalent pathogroups (FEP), where pathogenic and commensal bacteria co-aggregate symbiotically in a pathogenic biofilm to maintain a chronic infection [7]. This polymicrobial biofilm has been observed both in pre-clinical studies using animal models and in clinical research on DFU. It represents the main cause of delayed healing.

## 2. Pathophysiology of Diabetic Foot Ulcers

### 2.1. Main Host-Related Factors

The triopathy induced by diabetes *mellitus* plays a role in the origin and chronicity of the DFU.

▪ **Diabetic immunopathy**: Diabetic patients have an altered function of polymorphonuclear cells and impaired phagocytosis, chemotaxis, and bactericidal activity (related to both non oxidative and oxidative mechanisms), which are more evident in the presence of high hyperglycemia [8]. A study on diabetic mice showed that persistent hyperglycemia had a deleterious effect on the innate immunity and could lead to skin and soft tissue infections by *Staphylococcus aureus* [9].
▪ **Diabetic neuropathy**: Neuropathy by C-fiber and autonomic nerve fiber dysfunction is a common and frequent complication of diabetes *mellitus*. An evolution of the deregulation of glycemic balance is the inhibition of nociception and the perception of pain, a process called loss of protective sensation [10]. Thus, patients may not initially notice small wounds in the legs and feet, and may fail to prevent infection. Studies have observed a reduction in foot skin innervation and the expression of neurogenic factors in DFU, correlated with low inflammatory cell accumulation and therefore in the chronicity of DFU. This contributes to enhancing susceptibility to infection of diabetic neuropathic foot ulcers [11].
▪ **Diabetic angiopathy**: Peripheral arterial disease (PAD) and microangiopathy are the main risk factors for DFU. The decrease in the oxygenation of tissues by thickening the capillary basement membrane is a hallmark of diabetic angiopathy [12]. Disease of arteries in the lower limb is a well-known risk factor for DFU. Indeed, studies have shown that PAD presents a 5.5-fold increased risk for DFU [13]. The ischemia caused by the angiopathy also enhances the severity of the infection as a result of a poor delivery of oxygen and nutrients in the infected wound and because of poor antibiotic tissue penetration [14].

Finally, the anatomical characteristics of the foot, with its division into compartments, participates in the pathophysiology by increasing the severity of the infectious process by promoting the spread of infection and aggravating tissue damage.

### 2.2. DFU Microbiota

The host–microbiota interface is often the key point in the development of wound infections. Defining the diabetic foot microbiota implies the possibility of distinguishing it from skin microbiota associated with other clinical statuses. Compared with the feet of non-diabetic men, those of diabetic men had decreased populations of *Staphylococcus* spp., increased populations of *S. aureus*, and increased bacterial diversity [15]. When compared with contralateral healthy skin, the DFU microbiota harbored less bacterial diversity with greater levels of opportunistic pathogens [16]. However, neither patient demographics nor wound type influenced the bacterial composition of the chronic wound microbiome [17]. Different studies have described this DFU microbiota [17,18,19,20,21,22,23,24,25]. Although they have produced interesting results and confirmed that the microbiota is a highly dynamic microbial community that maintains a relationship with the host, understanding the complex competitive or synergistic interaction between commensal and pathogenic microorganisms is necessary as it could play an important role in the severity and evolution of the wound.

### 2.3. Disturbances in the Host–Microorganism Interplay

▪ **Bacterial virulence**: The virulence of pathogens is a key element in the pathophysiology of DFU. The ability of a bacterium to be virulent is key to the precarious balance between colonization and infection [26]. Bacterial virulence has been characterized using DNA microarray-based genotyping, multiplex polymerase chain reaction (PCR), and in vivo assays [26,27]. Among the large panel of virulence factors, bacterial proteases (serine-, cysteine-, and metallo-proteases), produced by a wide range of pathogenic bacteria, could play a major role in the pathogenesis of wound healing [28]. However, these wounds, and especially DFU, are highly polymicrobial, and bacterial interactions should also be studied in order to better understand the mechanisms of infection and the role of each of the pathogens involved in DFU.
▪ **Biofilm organization**: In a 2008 study assessing wound tissue biopsies using electron microscopy, James et al. suggested that 60% of chronic wounds present biofilms versus 6% for acute wounds [29]. In the following sections of this review, we focus on the formation of biofilms, evidence of biofilms in DFU, influence of the diabetic environment, and finally the clinical implications of biofilms in DFU.

## 3. Overview of Biofilms in DFU

### 3.1. Biofilm Formation in DFU and Tools for Detection

**Figure 1:**
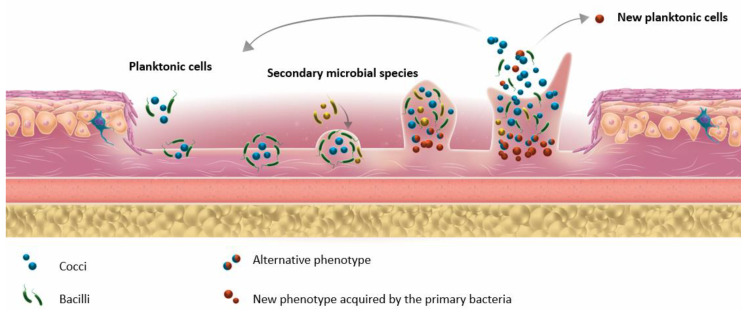
Different bacterial steps of biofilm formation.

In the environment, microorganisms can exist in two main states, namely: planktonic and sessile. In the planktonic state, bacteria move freely in their environment. In the sessile state, microorganisms are attached either to solid surfaces (e.g., urinary catheter or prosthesis), or more frequently, to each other, constituting multicellular aggregates that can lead to biofilm formation. Biofilm formation is a multistep process (for review, see Percival et al., 2015 [30]; Figure 1) whereby heterogeneous communities of microorganisms (bacteria and/or fungi) [30] are embedded into a self-produced matrix of extracellular polymeric substance (EPS). EPS contains proteins, glycoproteins, and polysaccharides and confers the ability to adhere to biotic or abiotic surfaces [31]. Clinically, biopsy tissues are the most reliable samples for revealing biofilms in deep tissues. However, the use of swabs to collect biofilm samples from the wound surface is considered an improper technic because of contamination from the skin microbiota, the difficulty in detaching the biofilm from the host epithelium, and the growth of anaerobes in the deep tissues. If a moderate to severe soft tissue infection is suspected and a wound is present, a tissue sample from the base of the debrided wound should be examined. Biofilms in tissue samples are commonly quantified through microscopy. Techniques such as confocal laser scanning microscopy and scanning electron microscopy or fluorescence in situ hybridization (FISH) are the most appropriate for revealing biofilms in biopsies [32].

Some important features of chronic wounds, and notably DFU, could be noted as follows:

▪ Cells included in the biofilm can develop an intracellular communication mechanism called quorum sensing (QS) [33], which controls bacterial pathogenicity and biofilm formation. The bacterial density influences the biofilm production [34].
▪ Microbial cells within a biofilm can detach and disseminate in the wound environment. The behavior of the released bacteria may differ from that of the pioneering colonizing bacteria because of adaptation within the biofilm [30,35].
▪ The concept of FEP was proposed by Dowd et al. after observing that different bacterial species can collaborate and interact with each other. FEPs are responsible for the chronicity of infection and for the maintenance of the pathogenic biofilm [7].

### 3.2. Biofilm Studies in Animal Models of DFU

Several studies have described the presence of biofilms in animal wounds since the early 2000s, and experimental diabetic models were developed in 2010 (Table 1). Pioneering groups in this field have shown that in db/db mice (a model of diabetic dyslipidemia), *Pseudomonas aeruginosa* or *S. aureus* biofilms delayed wound healing, and that the diabetic condition slowed down healing and increased the biofilm thickness [36,37]. Hsu et al. also reported that high glucose levels encourage the formation of vancomycin-resistant *S. aureus* biofilms [38]. Other studies have shown that the host response and neutrophil oxidative burst activity were decreased in the wound, and that oxidative stress and reactive oxygen species promoted biofilm appearance [39,40]. James et al. suggested that biofilms in wounds induced oxygen-limiting conditions (and thus stress) by the following two mechanisms: (i) bacterial metabolic activities and (ii) oxygen-deprivation by the host defenses [41]. These findings were recently confirmed by Hunt et al., who showed delayed healing in diabetic mice concomitantly with increased pus production [42]. They also suggested that in db/db mice, the deleterious impact of *P.* *aeruginosa* on wound healing cannot be explained solely by its ability to form biofilms, and that the type-3 secretion system virulence structure was also involved in the wound damage caused by this pathogen [43] (Table 1).

### 3.3. Biofilm Studies in Human Clinical DFU

Many clinical studies emerged in the 2010s demonstrating the impact of biofilms in chronic wounds (Table 2). In 2011, Neut et al. published two case studies of diabetic patients with non-healing ulcers. Using the confocal laser scanning microscope technique, they showed evidence of biofilms in diabetic wounds [44]. Subsequently, several studies have shown the presence and the impact of biofilms in clinical DFU. Malik et al. showed that on 162 diabetic foot infections (DFI), biofilms were present in 67.9% of the cases [45]. Other studies supported this and, in particular, the implication of *S. aureus* within the biofilms [46,47]. Oates et al. confirmed the importance of biofilms, using 26 human samples after debridement, employing FISH and scanning electron microscopy [48]. Recent research has shown that, during infection, in particular at the wound level, a single bacteria species is not responsible for biofilm formation [49]. Instead, microbes represent a complex polymicrobial biofilm community communicating with each other [50]. Interactions between microbes are complex and play an important role in the pathogenesis of the infection. These interactions range from competition for nutrients to evolving cooperative mechanisms that support their mutual growth in a specific environment [51]. Proximity and contact between bacteria in the biofilm promote communication and exchanges. To adapt their behavior, bacteria communicate through diffusible molecules like homoserines lactones or quinolones for Gram-negative bacteria, whereas Gram-positive cocci use short peptides [52]. Moreover, this proximity contributes to horizontal gene transfer, providing tolerance to antimicrobial agents and enhancing survival. Mottola et al. studied 53 staphylococci clinical isolates from DFU [53]. They discovered that biofilms cells were 10 to 1000 more tolerant to antibiotics than planktonic cells. In their work, of the 10 antibiotics tested, only gentamicin and ceftaroline were able to eradicate the biofilms. It has been reported that bacterial biofilms are also highly resistant to ultraviolet and heavy metals [54]. In addition to bacteria, fungi, especially *Candida*, are present in DFU biofilm-associated wound samples [55].

### 3.4. Factors Influencing Biofilm Formation in DFU

DFUs are mainly colonized by commensal bacteria. Numerous papers have analyzed the DFU microbiome, showing that the wounds contain commensal microorganisms from different niches [57,58]. All of these studies highlight the high bacterial complexity of wounds. This complexity is one of the major characteristics of DFU, and the lack of knowledge regarding the interactions of these microorganisms in the wound renders these infections as being complicated to manage [59]. The microorganisms appear to be organized as multi-layered communities surrounded by a self-produced protective extracellular matrix, and are organized into different FEPs [7]. Biofilm formation is a multistep process, including random settlement of early bacterial colonizers, with increased competition among species and niche differentiation, resulting in highly heterogeneous biofilms [30]. The biofilms detected in patients with foot ulcers may be responsible for the delayed healing of these chronic wounds [18]. Moreover, the presence of some bacterial communities in the initial stages of the wounds has been associated with delayed healing [24].

Several microbial and host factors specific to DFU may interfere in the development and feature of the biofilms:

- High bacterial diversity [7,15,60,61], including opportunistic pathogens [16] and anaerobic bacteria [57,62].
- Increased *S. aureus* population [15], particularly in neuropathic DFUs [61]. However, their microbiota present a similar level of richness (number of different species in the wound community), abundance, and diversity compared to other chronic wounds [63], suggesting that the microbiota is not influenced by the wound type.
- The wound depth with a more diverse and complex microbiota in the deep part of the wound [64] where pathogenic, particularly anaerobic, bacteria are sheltered.
- Environmental factors (e.g., demographic characteristics, personal hygiene, geographical location of the patient, high glycemic level, and previous exposure to antimicrobial therapy) [65].
- Patient immune status that modifies the role of low-virulence bacteria (e.g., *Staphylococcus* sp. and corynebacteria) towards a higher pathogenicity [66], and where excessive secretion of pro-inflammatory cytokines, pH, temperature, or antimicrobial treatment (topic or systemic administration) [67] can increase tissue destruction [68].
- DFU duration is positively correlated with the ecological diversity of the bacteria present in the wounds, species richness, and relative abundance of *Proteobacteria*. It is also negatively correlated with the relative abundance of staphylococci [69].
- Local tissue hypoxemia is often observed as a result of obstructive arteriopathy. This hypoxic environment influences bacterial diversity, with a higher prevalence of proteobacteria and strict anaerobic bacteria in deeper ulcers [61,68].
- The development of a “unique microbiota” in each DFU (new or recurrent) [17].

### 3.5. Bacterial Organization Inside DFU

The main characteristic of DFU is the polymicrobial content that modulates bacterial virulence. Within DFU, microorganisms form a complex polymicrobial biofilm community and intercommunicate [7]. As described above, bacterial interactions play an important role in pathogenesis, competing and cooperating in order to support their mutual growth in a specific environment [51] via interactions through diffusible molecules [52].

The most studied bacterial interaction in DFU is the cooperation between *S. aureus* and *P. aeruginosa*, despite the location of *P. aeruginosa* being deeper in the wound bed than *S. aureus* [70]. Many substances produced by *P. aeruginosa* may play a protective role for *S. aureus* [17,70,71,72,73,74]. In a rat model of orthopedic wounds, even a low presence of both *P. aeruginosa* and *S. aureus* increased their infection rates in the wound [75]. A similar synergistic cooperation between *P. aeruginosa* and *S. aureus* also increased their tolerance to antibiotics, ability to form biofilms, and the secretion of virulence factors (hydrogen cyanide, exoenzyme S, exotoxin A, and pyocyanin for *P. aeruginosa*, and Panton-Valentine leukocidin and α hemolysin for *S. aureus*) [76]. These interactions can also be competitive, as exemplified by the competition for iron or the one-way growth inhibition of *S. aureus* [37,77]. Indeed, *P. aeruginosa* can simultaneously suppress *S. aureus* growth and enhance its resistance to aminoglycosides [71].

Other bacterial interactions have also been described. For instance, the combined inoculation of different pathogenic bacteria (*Escherichia coli, Bacteroides fragilis*, and *Clostridium perfringens*) increased the mortality rate in type-2 diabetic mice compared with those receiving inoculation of single strains [78]. Competition between commensal and pathogenic bacteria has been observed during cutaneous colonization [79]. In contrast, *Helcococcus kunzii* (a commensal Gram-positive coccus) and *S. aureus* cooperation led to a decrease of *S. aureus* virulence *in Caenorhabditis elegans* [80]. *S. aureus* shifts toward commensalism in response to *Corynebacterium* sp. [81]. Moreover, *S. epidermidis*, a commensal bacterium, produces a serine protease (Esp) that inhibits *S. aureus* biofilm formation [56,82]. Finally, the co-culture of *Fusobacterium nucleatum* (ATCC 25586) with *Prevotella intermedia/Prevotella nigrescens* promotes biofilm formation compared with single cultures [83].

Another pertinent aspect of polymicrobial biofilms in DFU is their ability to adapt under various circumstances via enhanced metabolic cooperation and gene regulation between sessile cells. Biofilm diversity promotes its survival by creating a thicker biofilm, resulting in more severe infections. In this context, Mottola et al. reported that the biofilms formed by *P. aeruginosa* and *Enterococcus faecalis* and *Acinetobacter baumannii,* and *S. aureus* resulted in a thicker biofilm than the bacteria alone, which were difficult to eradicate [84]. Furthermore, these microbial communities are heterogeneous. Interestingly, fungi can also form biofilms. Both yeasts and filamentous fungi can adhere to biotic and abiotic surfaces, and form highly organized communities that are resistant to antimicrobials and environmental conditions. Many fungi have been correlated with biofilm formation, however, *Candida* biofilms remain the most widely studied. The biofilms formed by yeast and filamentous fungi present differences, and studies of polymicrobial communities have become increasingly important. Interactions have been observed between bacterial and fungal species in chronic wounds [55]. Infections that are thought to involve polymicrobial biofilms are most frequently associated with the abiotic surfaces of indwelling medical devices. In a review written by Lynch and Robertson [85], they highlighted the indwelling medical devices commonly associated with biofilm formation. In all of the devices tested, the principal pathogen responsible for the biofilms was a bacterium, however in 70% of cases, fungi was found as a secondary species. Among fungal pathogens, *Candida albicans*, a commensal mucosal organism and opportunistic pathogen of the immunocompromised, was most commonly associated with biofilms. Numerous studies have described co-infections of fungi and bacteria in different diseases. For example, cystic fibrosis lungs are a major site of polymicrobial infection, with bacteria such as *P. aeruginosa, S. aureus, Burkholderia cepacia, A. baumannii,* and *Haemophilus influenzae* mixed with *C. albicans, A. fumigatus*, and *Scedosporium* sp. [86]. In a DFI context, Kalan et al. showed that the presence of fungal communities in the polymicrobial biofilms of chronic wounds is associated with a poor prognosis and delayed healing [87]. Further studies are needed in order to fully elaborate on the role of each microorganism in the polymicrobial biofilms of DFU.

## 4. Clinical Impact of Biofilms in DFU

As biofilms are implicated in 60 to 80% of chronic wounds [29,88], the clinical impact of biofilms is particularly relevant. For clinicians, the main difficulty is in distinguishing between infecting and colonizing bacteria. Misidentification can lead to inappropriate antibiotic prescriptions that may contribute to the emergence of multidrug resistant (MDR) bacteria, a major DFU health issue [30].

### 4.1. Antibiotics Resistance

Sessile cells involved in biofilm formation display different characteristics compared with non-biofilm-associated cells (i.e., planktonic cells) [89]. In particular, sessile cells show a higher tolerance towards antimicrobial agents, one of the main causes of treatment failure [90,91]. Antimicrobial agent tolerance arises by several mechanisms, namely: (i) inability of drugs to penetrate through the polymeric matrix; (ii) the lack of intracellular accumulation of antibiotics due to impermeability (e.g., excessive production of glucans by *P. aeruginosa*) or active efflux (e.g., increased expression of efflux pump genes in Gram-negative bacilli); (iii) the presence of sessile bacteria, whereby cells are metabolically inactive and thus tolerate the antibiotic action better; and (iv) the importance of horizontal gene transfer between bacteria for the diffusion of resistant traits [92,93]. Biofilms increase the opportunity of gene transfer of virulence factors and antibiotic-resistant genes to susceptible bacterial species. The rate of mutation occurring in biofilms is markedly higher compared with planktonic cells [94]. In addition, (v) stress response to hostile environmental conditions (e.g., leading to an overexpression of antimicrobial agent-destroying enzymes) can result in an altered microenvironment inside the biofilm matrix (pH and oxygen content) and may contribute to enhanced degradation of antimicrobial agents in the biofilm matrix [95]. Finally, the hypoxic environment present in DFU also modulates the tolerance of bacteria to some antibiotics. For instance, the in vitro bactericidal effect of vancomycin on *S. aureus* isolates is lower in anaerobic conditions [96].

### 4.2. Host Immune Response

Pioneering colonizing bacteria released from the biofilm can adapt to their environment and form a new biofilm. To our knowledge, the only study conducted in this field focused on *Klebsiella pneumoniae* [35]. In addition, EPS is a mechanical barrier to antimicrobials, as well as to immune system cells [97]. Bacteria within biofilms evade the host’s natural defenses and are resistant to the host immune defense by different mechanisms, including the following: (i) limited penetration of leukocytes and their products into the biofilm [98]; (ii) global response regulators and quorum sensing, which protect the biofilm bacteria [99]; (iii) decreased phagocytic capacity of host cells against biofilm bacteria [100]; (iv) genetic switches that increase the resistance of biofilm bacteria [101]; and (v) suppression of the leukocyte effector function, including softening the magnitude of the respiratory burst [102]. Indeed, stimulation of the immune system without effectively eradicating the infection causes collateral damage to surrounding tissue and causes chronic inflammation [103]. This persistent chronic inflammation, added to the diabetic immune context, leads to the production of auto-inflammatory cytokines that aggravate the wound and slow the healing process.

## 5. Therapeutic Perspectives

Biofilms have a crucial role in DFU and DFIs and contribute to delayed healing. They are especially difficult to treat using classical antibiotics because of EPS, which prevents diffusion into the biofilm. They also support gene transfer, the selection of strains with beneficial characteristics, and the development of new bacterial characteristics. This difficulty in treating DFU/DFI could be enhanced in the context of the diabetic environment.

Biofilms encountered in chronic wounds, such as DFU, are highly polymicrobial, which can enhance bacterial interactions. Bacterial cooperation is key to understanding the formation and regulation of biofilms at a wound level, but also for highlighting new therapeutic targets. The available approaches against biofilms are quite limited, and new prevention, diagnosis, and treatment methods are crucially needed, particularly because of the extent of the MDR bacteria in this pathology.

Targeting biofilm formation could be an interesting strategy to prevent or at least reduce this problem. Classically, clinicians reduce the bacteria load (constituting commensal and pathogen species) resulting from the biofilm organization and FEP. The best method involves physical removal, also called debridement, of the infected tissue in order to improve healing [104,105]. It is often performed using surgical instruments or by irrigation [105], and is the initial and essential stage in the management of infected wounds. This strategy is still the preferred method used to prepare the wound bed and to promote moist wound healing, but it might not completely remove the biofilms immediately. Therefore, it must be repeated at regular intervals [104]. The results obtained with ultrasound debridement could represent a promising approach [106]. Other approaches could be proposed with the aim to inhibit bacterial adhesion or biofilm metabolism, such as (i) blocking bacterial adhesins (using ions chelators such as ethylenediaminetetraacetic acid (EDTA) and citrate, the most promising compounds of this class [107]), (ii) inhibiting the adhesion structure biogenesis (e.g., plant-derived natural compounds [108]), (iii) modulating QS (e.g., furanone [109], savarine [110], or deferiprone [111]), and (iv) enhancing bacterial dispersion (such as the α-amylase enzyme [112], 2-aminoimidazole [113], or Cis-2-decenoic acid [114]). Physical inhibition could also represent an interesting method, such as photodynamic therapy-induced pathogen cell death by killing sessile bacteria [115].

Some antimicrobial strategies as alternatives to antibiotics have also be developed, such as phagotherapy [116,117], nanotechnologies [118], antimicrobial peptides (AMP) [119,120,121], or agent mimicking AMPs [122], as well as natural compounds (such as honey [123]). These approaches have an interesting potential, but further studies are required to really understand the mechanism of action of each of these solutions and to improve their role in DFI management.

Researchers are now aware of and consider the polymicrobial characteristics of DFI and biofilms. Further studies on bacterial interactions are required in order to really understand the pathophysiology and to help with the development of new therapeutic tools that will target polymicrobial biofilms. This needs to be done through the development of (i) validated, consistent, and robust animal wound models reproducing the clinical situation and biofilm constitution; (ii) ex vivo and in vivo imaging technologies to visualize bacterial biofilms and to confirm their eradication; and (iii) “omics” tools to detect biofilm formation at the bedside and to evaluate the best course of action for the debridement.

## 6. Conclusions

Biofilms have a crucial role in DFIs and contribute to delayed healing. These wounds are characterized by a complex microbiome and a polymicrobial organization, especially within the biofilm. Even if most experimental biofilm studies provide descriptive and interesting information, they are derived from in vitro studies or non-adapted in vivo models. The development of processes and methodologies to study biofilms is needed. This represents the next step to validating new antibiofilm molecules with a promising therapeutic potential.

## Figures and Tables

**Table 1:** Examples of biofilm studies in animal models of diabetes.

Animal Model	Strain Used	Findings	Reference
db/db mice	*P. aeruginosa* (PAO1)	Biofilm evidence after a 6-mm punch biopsy wound on the dorsal skin	[36]
db/db mice	*P. aeruginosa* (PAO1)	Biofilm delays wound healing	[37]
TallyHo mice (Type 2 diabetes *mellitus*)	*P. aeruginosa*	Biofilm decreases TLR 2, TLR 4, IL-1α, and TNF-α expression and neutrophil oxidative burst activity	[39]
BALB/c mice with injection of STZ	Vancomycin-resistant *S. aureus*	Correlation between glucose concentration and biofilm formation	[38]
db/db mice	Wound microbiome	Oxidative stress and ROS favor biofilm formation and establish a chronic wound	[40]
db/db mice	*P. aeruginosa*	Bacteria in biofilm induce oxygen stress by producing metabolites and recruiting defense cells that reduce oxygen	[41]
Mice with injection of STZ	*P. aeruginosa*	Biofilm increases wound depth, mortality rate, and pus production	[42]
db/db mice	*P. aeruginosa*	*P. aeruginosa* infection is independent of its ability to form biofilm and primarily depends on T3SS	[43]

db/db mice—diabetic mice; TLR—toll-like receptor; IL—interleukin; TNF—tumor necrosis factor; ROS—reactive oxygen species; STZ—streptozocin (a pancreatic β-cell toxin); T3SS—type-3 secretion system.

**Table 2:** Examples of biofilm studies in clinical human DFU.

Model	N° of Patients	Biofilm Visualization	Findings	Reference
DFU	2	CLSM	Evidence of biofilms	[44]
DFU	162	Microtiter plate assay	Biofilms in 67.9% of infected DFUs	[45]
DFU	26	FISH and ESEM	Observation of the formed biofilms and their bacterial constitution	[48]
DFU	357	Crystal violet	Observation of the formed biofilms	[46]
DFU	100	Congo Red dye, tissue culture plates, and crystal violet staining	Biofilm formation in 46.3% of isolates, predominantly by *S. aureus* (38.8% of isolates) and MDR bacteria (46.3%)	[47]
DFU	49	Calgary biofilm pin lid device with resazurin and PCR of genes associated with biofilm formation	Biofilms are resistant to antibiotics at concentrations 10–1000 times higher than those required to kill planktonic cells	[53]
DFU	155	Microtiter plate assay and ELISA, XTT formazan, and SEM	Presence and importance of non-*Candida albicans* species in biofilms	[55]
DFU	95	Microtiter plate assay and FISH	Polymicrobial biofilms are thicker	[56]

DFU—diabetic foot ulcer; CLSM—confocal laser scanning microscopy; ELISA—enzyme-linked immunosorbent assay; ESEM—environmental scanning electron microscopy; FISH—fluorescent in situ hybridization; MDR—multidrug resistant; PIA—polysaccharide intercellular adhesin; SEM—scanning electron microscopy; XTT—2H-tetrazolium-5-carboxanilide.
